# Cost-Effectiveness of the Interventions for Severe Mental Illness (SMI) in Low and Middle-Income Countries (LAMICs): A Systematic Review

**DOI:** 10.1192/bjo.2025.10170

**Published:** 2025-06-20

**Authors:** Abdul Latif, Martyn Lewis, Ghasem Yadegarfar, Zala Khan, Saeed Farooq

**Affiliations:** 1 School of Medicine, Keele University, Newcastle Under Lyme, United Kingdom; 2 Institute of Public Health and Social Sciences, Khyber Medical University, Peshawar, Pakistan

## Abstract

**Aims:** Despite the high prevalence of mental illnesses, particularly Severe Mental Illness, there is limited literature on the cost-effectiveness of available interventions in low- and middle-income countries. Therefore this review aimed to assess the cost-effectiveness of the pharmacological and psychosocial interventions for Severe Mental Illness (SMI) in Low and Middle-Income Countries (LMICs).

**Methods:** Based on PRISMA guidelines through electronic searches (Medline, CINAHL, APA PsycINFO, Embase, Cochrane Central Register of Controlled Trials, and Global Index Medicus), we identified cost-effectiveness studies conducted between January1980 and April 2024. Studies included whether they focused on people with schizophrenia, bipolar disorders and depression with psychosis, assessed any interventions (pharmacological or psychosocial), and reported cost-effectiveness outcomes based on predefined criteria, specifically presented as incremental cost-effectiveness ratio (ICER) values. Screening and data extraction were performed using a pre-specified criterion. ECOBIAS and JBI tools were used for quality assessment.

The analysis was confined to a narrative synthesis due to the substantial variations in methods adopted for ICER calculations and the inherent complexity of model-based studies. Protocol was registered on the PROSPERO having registration # *CRD42024513743.*

**Results:** Out of the 6905 studies identified, 20 met the inclusion criteria for data extraction. Most of the studies (18/20) were based on the economic model, predominantly the Markov Model (11/18). Most of the studies were conducted in upper-middle-income countries (13/20). Atypical or second-generation antipsychotics were the major group evaluated in most of the studies. The cost-effectiveness of olanzapine was assessed in the highest number of studies (10/20), followed by risperidone. “Family intervention” was the predominant psychosocial intervention and was evaluated in three studies. Ten studies reported ICER in terms of cost/QALYs gained, while 6/20 studies reported cost/DALYs averted. The remaining studies assessed cost-effectiveness in the context of cost savings against the Positive and Negative Symptoms scale.

Cost-effectiveness was evaluated based on the quadrants of the cost-effectiveness plane in which the ICER values fell. In upper-middle-income countries, atypical such as amisulpride, lurasidone, aripiprazole orally disintegrating tablets (ODT) and olanzapine with any psychosocial interventions were cost-effective strategies. In contrast, risperidone with Family Interventions was reported as the cost-effective strategy in lower-middle-income countries.

**Conclusion:** Most studies have found that combining atypical antipsychotics with psychosocial interventions is a cost-effective approach. However, significant variations in ICER calculations, differences in methods used to assess QALYs/DALYs, and the complexity of model-based studies make it challenging to generalize these findings to other clinical settings.

